# Titanium-doped phosphate glasses containing zinc and strontium applied in bone regeneration

**DOI:** 10.1007/s10856-024-06804-z

**Published:** 2024-06-20

**Authors:** Tianyi Tang, Rachel Wandless, Zalike Keskin-Erdogan, Nandin-Erdene Mandakhbayar, Jeong-Hui Park, Hae-Won Kim, Morgana Abramchuk, Felipe P. Daltoe, Jonathan C. Knowles

**Affiliations:** 1 Division of Biomaterials and Tissue Engineering, UCL Eastman Dental Institute, University College London, Royal Free Hospital, Rowland Hill Street, London, NW3 2PF UK; 2 Department of Chemical Engineering, Imperial College London, Exhibition Road, South Kensington, London, SW72AZ UK; 3 Institute of Tissue Regeneration Engineering (ITREN), Dankook University, Cheonan, 31116 South Korea; 4 UCL Eastman-Korea Dental Medicine Innovation Centre, Dankook University, Cheonan, 31116 South Korea; 5 Department of Nanobiomedical Science & BK21 NBM Global Research Centre for Regenerative Medicine, Dankook University, Cheonan, 31116 South Korea; 6 Department of Biochemistry, School of Biomedicine, Mongolian National University of Medical Sciences, Ulaanbaatar, Mongolia; 7 Department of Biomaterials Science, College of Dentistry, Dankook University, Cheonan, 31116 South Korea; 8 Graduate Program in Dentistry, Federal University of Santa Catarina, Florianopolis, SC 88040-370 Brazil; 9 Department of Pathology, Federal University os Santa Catarina, Florianopolis, SC 88040-370 Brazil

## Abstract

Phosphate bioactive glass has been studied for its advanced biodegradability and active ion release capability. Our previous research found that phosphate glass containing (P_2_O_5_)–(Na_2_O)–(TiO_2_)–(CaO)–(SrO) or (ZnO) showed good biocompatibility with MG63 and hMSCs. This study further investigated the application of 5 mol% zinc oxide or 17.5 mol% strontium oxide in titanium-doped phosphate glass for bone tissue engineering. Ti-Ca-Na-Phosphate glasses, incorporating 5% zinc oxide or 17.5% strontium oxide, were made with melting quenching technology. The pre-osteoblast cell line MC3T3-E1 was cultured for indirect contact tests with graded diluted phosphate glass extractions and for direct contact tests by seeding cells on glass disks. The cell viability and cytotoxicity were analysed in vitro over 7 days. In vivo studies utilized the tibial defect model with or without glass implants. The micro-CT analysis was performed after surgery and then at 2, 6, and 12 weeks. Extractions from both zinc and strontium phosphate glasses showed no negative impact on MC3T3-E1 cell viability. Notably, non-diluted Zn-Ti-Ca-Na-phosphate glass extracts significantly increased cell viability by 116.8% (*P* < 0.01). Furthermore, MC3T3-E1 cells cultured with phosphate glass disks exhibited no increase in LDH release compared with the control group. Micro-CT images revealed that, over 12 weeks, both zinc-doped and strontium-doped phosphate glasses demonstrated good bone incorporation and longevity compared to the no-implant control. Titanium-doped phosphate glasses containing 5 mol% zinc oxide, or 17.5 mol% strontium oxide have promising application potential for bone regeneration research.

**Graphical Abstract:**

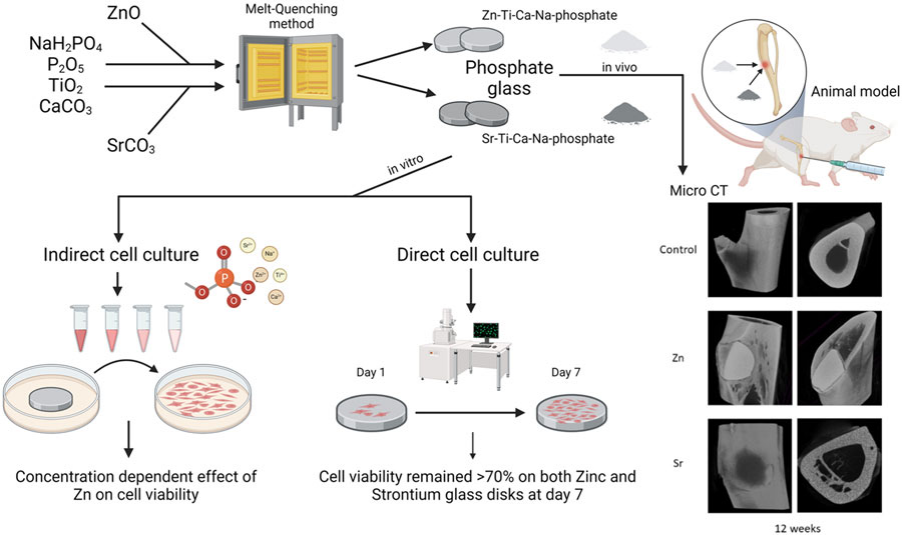

## Introduction

Bioactive glass is a promising biomaterial that exhibits several favorable characteristics in bone tissue engineering [1]. It continuously attracts attention due to its excellent mechanical properties and good biocompatibility. Silicate-based bioactive glasses have been widely studied, with several clinical trials being performed using its commercial products [2]. It can promote bone formation, increase the density of bone defects, and is compatible with autogenous bone grafts [3–5]. However, the application of silicate bioactive glass is still limited by its insufficient biodegradability.

Phosphate bioactive glass has been studied for advanced biodegradability and active ion release. Its glass network is formed by P_2_O_5_ and can be modified by other components like calcium dioxide and sodium oxide [6]. Due to the inherent similarities between phosphate glass and bone tissue, this biomaterial naturally possesses the potential for applications in bone repair. Unlike some inert materials, a major advantage of phosphate glass is that it has active effects, such as ion release. More importantly, titanium oxide has been found to significantly improve the stability of phosphate glass when its component is between 3 mol% and 7 mol% [7]. This finding is highly encouraging, as this property facilitates controlled biodegradation, avoiding destructive outcomes associated with pH changes and ion release.

Multiple metal oxides can be processed in a phosphate glass system for specific applications. The ion release of zinc is also good for promoting proliferation and anti-bacterial effect [8, 9]. However, it was reported that increasing zinc oxide to 15%mol leads to a significant cytotoxic effect. The application range of zinc in phosphate glass disks is around 5–10 mol% according to previous research [10].

Strontium-doped phosphate glass has the potential for active ion delivery and promotion of bone formation [11, 12]. Previous research succeeded in making phosphate glasses with up to 5 mol% of strontium which showed low cytotoxicity [13]. The following research investigated up to 35 mol% of strontium in phosphate glass and found that 17.5 mol% of strontium exhibited promising biocompatibility [14].

In this study, we want to further study the application of 5 mol% of zinc oxide or 17.5 mol% of strontium oxide in titanium phosphate glass for bone regeneration. All the samples were prepared based on melting quenching technology. In vitro studies were performed to test the biocompatibility and an in vivo study was performed to detect bone formation in rats. We hypothesize that phosphate glasses containing 5 mol% zinc oxide, or 17.5 mol% strontium oxide exhibit low cytotoxicity and good biocompatibility for bone tissue engineering applications.

## Materials and methods

### Sample preparation

Phosphate glass with either 5% zinc or 17.5% strontium was made using the composition described in Table 1. The procedures include phosphorus pentoxide (P_2_O_5_, ≥98.0%, 21410.293, VWR), calcium carbonate (CaCO_3_, ≥99%, 22296.294, VWR), sodium dihydrogen phosphate (NaH_2_PO_4_, ≥98.0%, 0571, VWR), titanium dioxide (TiO_2_, ≥99.0%, 20732.298, VWR), zinc oxide (ZnO, ≥99.0%, 96479, Sigma–Aldrich), and strontium carbonate (SrCO_3_, ≥99.0%, 96479, Sigma–Aldrich). These components were mixed with the P_2_O_5_ added last due to its hygroscopic properties, and then transferred into a clean platinum crucible. The crucible was placed in a preheated furnace (Carbolite) at 700 °C for 30 min to remove the CO_2_ and H_2_O and then the temperature was raised to 1300 °C for 3.5 h. The molten glass was then poured into a preheated mold (diameter = 15 mm) at 420 °C to cool before being stored as glass rods. Consequently, the glass disks were sliced from a glass rod with 2 mm thickness.

**Table 1:** Compositions of Zn-Ti-Ca-Na and Sr-Ti-Ca-Na phosphate glass samples

	P_2_O_5_ (mol%)	Na_2_O (mol%)	TiO_2_ (mol%)	CaO (mol%)	ZnO (mol%)	SrO (mol%)
Zn-Ti-Ca-Na-phosphate glass	50	10	5	30	5	0
Sr-Ti-Ca-Na-phosphate glass	50	10	5	17.5	0	17.5

### SEM and energy dispersive X-ray spectroscopy (EDX)

Disc-only samples were oven-dried before being stuck on SEM specimen stubs. Samples that were seeded with cells were fixed in 3% glutaraldehyde and were dried with graded concentrations of ethanol (50–100%) followed by hexamethyldisilazane [7]. Samples were then stuck to SEM specimen stubs and coated in gold (80%) and palladium (20%) using an SEM coating unit (Polaron Equipment Limited). Samples were imaged using FE-SEM (Zeiss GeminiSEM) at EHT 10.00 kV. Energy Dispersive X-ray spectroscopy was performed with ZEISS SmartEDX in disc-only samples. The distribution of phosphorus, calcium, sodium, titanium, strontium, and zinc was mapped at EHT 20.00 kV.

### X-ray diffraction (XRD)

Phosphate glass disks containing either 5% zinc or 17.5% strontium were ground into powder using a vibrating sieve shaker (Analysette 3 Spartan, Fritsch). X-ray diffraction analysis (XRD) was performed using a Bruker D8 Advance Diffractometer (Bruker) in flat plate geometry using Ni-filtered Cu Kα radiation. Data were collected from 10–100° 2ϑ in flat plate geometry using Ni-filtered Cu Ka radiation and a step size of 0.019967° and a count time of 0.1 s. A Lynx Eye detector was used.

### Cell culture

Cells used throughout this project were pre-osteoblast MC3T3-E1 cell lines (99072810, Sigma Aldrich). Cells were cultured in T75 flasks in media composed of Minimal Essential Medium (11095080, Gibco), 10% Fetal Bovine Serum (26140079, Gibco) and 1% Penicillin- Streptomycin (15140122, Gibco). Cells were incubated at 37 °C and 5% CO_2_. To keep the growth and status of cells, the morphology and confluency of cells were checked, and the cell culture media was changed every 2 days. Cells were passaged once a week when 80% confluency was reached using Trypsin and subcultured into new flasks at a 1:8 split ratio. Before seeding MC3T3-E1 cell lines to microplates, cells were stained using trypan blue (Gibco) and counted using Neubauer Improved 0.1 mm Haemocytometer (Neubauer). To test cytotoxicity with extracts and direct contact, the seeding density is 10,000 cells per well in 96 well plates and 200,000 cells per well in 24 well plates, respectively. The extracts from the glass disc were prepared by incubating samples in 1 mL growth medium at 37 °C and 5% CO_2_ for 24 h. For direct contact, the cells were seeded on the surface of glass disks and well plates with low adherence surface were used to prevent growth in the bottom.

### Alamar blue

Alamar blue cell viability reagent (DAL1100, Invitrogen) is reduced in metabolically active cells causing it to turn red. This is then detected by fluorescence which is a direct indicator of the number of metabolically active cells. Before performing the Alamar blue assay, media was removed to be used for measuring LDH release. This media was then replaced with fresh media and 10% Alamar Blue. The cells were incubated at 37 °C and 5% CO_2_ for 2 h. 100 μl of media was removed and placed into a clear bottom opaque well plate. Fluorescence was detected using a BioTek FLx800 fluorescence microplate reader (Bio-Tek Instruments) at 560/590 nm. Percentage viability was calculated by dividing the fluorescence by the 2D control result x100.

### Lactate dehydrogenase (LDH) assay

LDH assays were performed to detect cytotoxicity following the manufacturer’s protocols from a commercial product (G7892, Promega). As the media was changed every other day, the LDH assays gave a snapshot of LDH release of 24 h before each time point. Besides, a lysis buffer was added to give a maximum LDH reading. The results of LDH release and maximum LDH release were collected from three replicates.

For LDH release, the growth media were collected at days 1, 3, and 7. Meanwhile, media was removed from the wells replaced with fresh media containing 1% Triton X-100 to detect maximum LDH release. Then, samples prepared for maximum LDH release were transferred into a 96-well plate alongside the media for the LDH release and the reaction mixture was added to all wells. The fluorescence intensity was measured at 560/590 nm after 10 min. For extracts, this was incubated in the dark at room temperature for 30 min. Absorbance was measured using the Tecan Infinite M200 microplate reader at 490 nm with a reference of 680 nm (C20300, Invitrogen). Each reading was taken three times and an average was created of the results. Percentage cytotoxicity was calculated by:

$$\% {cytotoxicity}=\frac{{LDH\; release}}{{Maxiumum\; LDH\; result}}x\,100$$

### Live and dead assay

The Live and Dead assay was performed to label the MC3T3-E1 cells that cultured on the surface of glass disks in 24 well plates for 1, 3, and 7 days. Media was removed thoroughly by washing with PBS buffer for 3 times. Cells were incubated with 0.2% ethidium homodimer and 0.05% calcein (L3224, Invitrogen) diluted in PBS buffer for 15 min at 37.5 °C and then being imaged under the inverted fluorescence microscope (Leica DM IRB).

### Fluorescence staining

Following the direct assay with phosphate glass disks, the cells were fixed with 10% formalin on days 1, 3, and 7. Then, samples were incubated with 1% Triton X-100 to permeate the cells, and 3% goat serum as a blocking buffer. After removing buffer, the samples were stained with Phalloidin (Abcam) and DAPI (Sigma–Aldrich) at 37 °C for 25 min. The disks were then transferred to glass bottom disks and imaged under the confocal microscope (Aurox mounted on Olympus BX51) using 10x and 20x water immersion lenses. Images were processed with Image J Fiji.

### Animal model

All animal procedures were conducted according to the protocol approved by the Ethics Committee on Animal Use of the Federal University of Santa Catarina (4148200917) and in accordance with the Brazilian National Council for Control of Animal Experimentation (CONCEA) guidelines. Rats were housed in groups of two or three per polycarbonate cage in a climate-controlled environment (21 ± 1 °C) with a 12 h light and dark cycle. Rats were randomly divided into three groups: I) Zinc (Zn2+); Strontium (Sr2+) and III) Control Group (no implants). A total of ten male Wistar rats (8 weeks old, 200–300 g body weight) had a single 2.5 mm diameter subcritical defect made in the distal aspect of the left tibia. Previously to the surgical procedure, animals received an analgesic drug (5 mg/kg Tramadol, subcutaneously) and a mixture of anesthetics comprising 100 mg/Kg Ketamine and 10 mg/kg Xylazine, intraperitoneal. The bone defect was created using a spherical bur (Jet Carbide Bur PM8), which had its active portion fully submerged into the bone. The defect was cleared with sterile saline irrigation previously to the phosphate glass implants. Biomaterials were dry heat sterilized and gently placed and packed into the bone defect. Immediately after the surgery, animals received a single intraperitoneal dose of Penicillin G 22.000 UI/Kg and ad libitum drinking water with Paracetamol (0.4 mg/Kg) for 3 days. The euthanasia procedure was carried out through deep anesthesia induced by an overdose of anesthetics, with doses of 20 mg/kg of Xylazine associated with 150 mg/kg of Ketamine. Additionally, cervical dislocation was performed as a method to ensure euthanasia.

### Micro-CT analysis

A rat in the control group was harvested immediately after surgery. Other rats were euthanized 2, 6, and 12 weeks after surgery. The left tibiae were harvested and fixed in 10% Formaldehyde. The bones were scanned with a Skyscan 1172 micro-CT scanner (Bruker, Coventry, UK), in small plastic tubes containing 70% ethanol. Scans were performed using 49KV beam energy and 200 μA flux, a 0.5 mm aluminum filter, and an isotropic pixel size of 5.5 μm. The medium pixel size used for scanning is 2000 × 1040.

### Statistics

All data are reported as averages calculated from triplicate results unless otherwise stated. Statistical was done using GraphPad Prism 9 software (GraphPad). Significance was assessed using a two-way ANOVA and Tukey’s multiple comparison test with a single pooled variance. Confidence level was set at 0.05 (95.5% confidence interval).

## Results

**Fig. 1:**
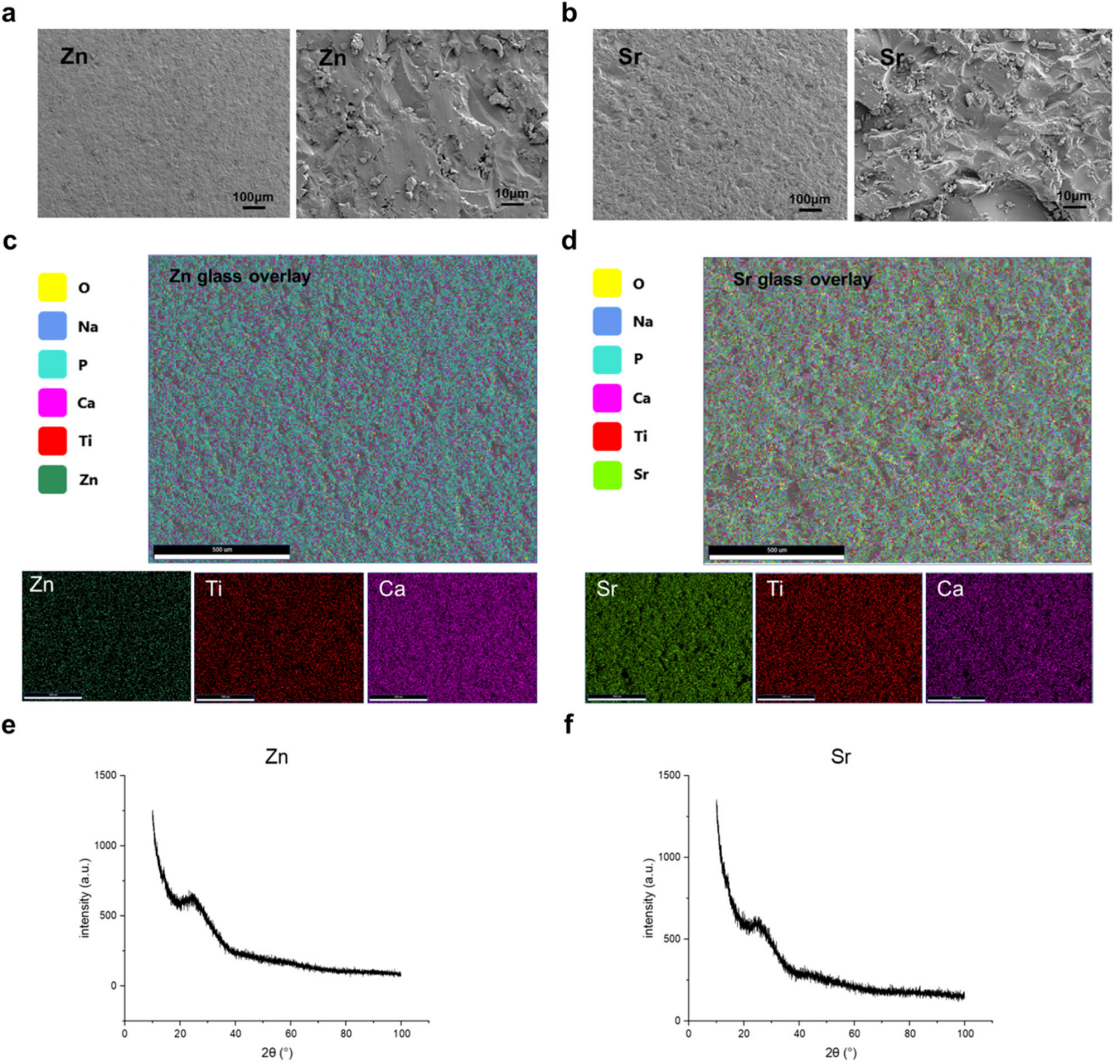
**a**, **b** Glass disks and SEM images at low and high magnification show relatively similar surface roughness for each of the materials. **c**, **d** EDX images showing the overlay of areas on the glass disks and the atomic percentage of different elements. Zn/Sr, Ti, and Ca image maps are also shown. **e**, **f** XRD analysis of Zn and Sr powdered glass samples

Two different titanium-doped phosphate glasses were successfully made with melting quenching technology. The glass rods with a diameter of 15 mm were prepared and then processed into glass disks for in vitro study. The SEM images showed the surface pattern of the Zn-Ti-Ca-Na-phosphate glass disc and Sr-Ti-Ca-Na-phosphate glass disc, respectively (Fig. 1a, b). The elements including phosphorus, calcium, sodium, titanium, oxygen, strontium, and zinc were shown and a homogeneous distribution of these elements was found in each sample (Fig. 1c, d). The XRD analysis from both samples showed a typical amorphous pattern (Fig. 1e, f).

**Fig. 2:**
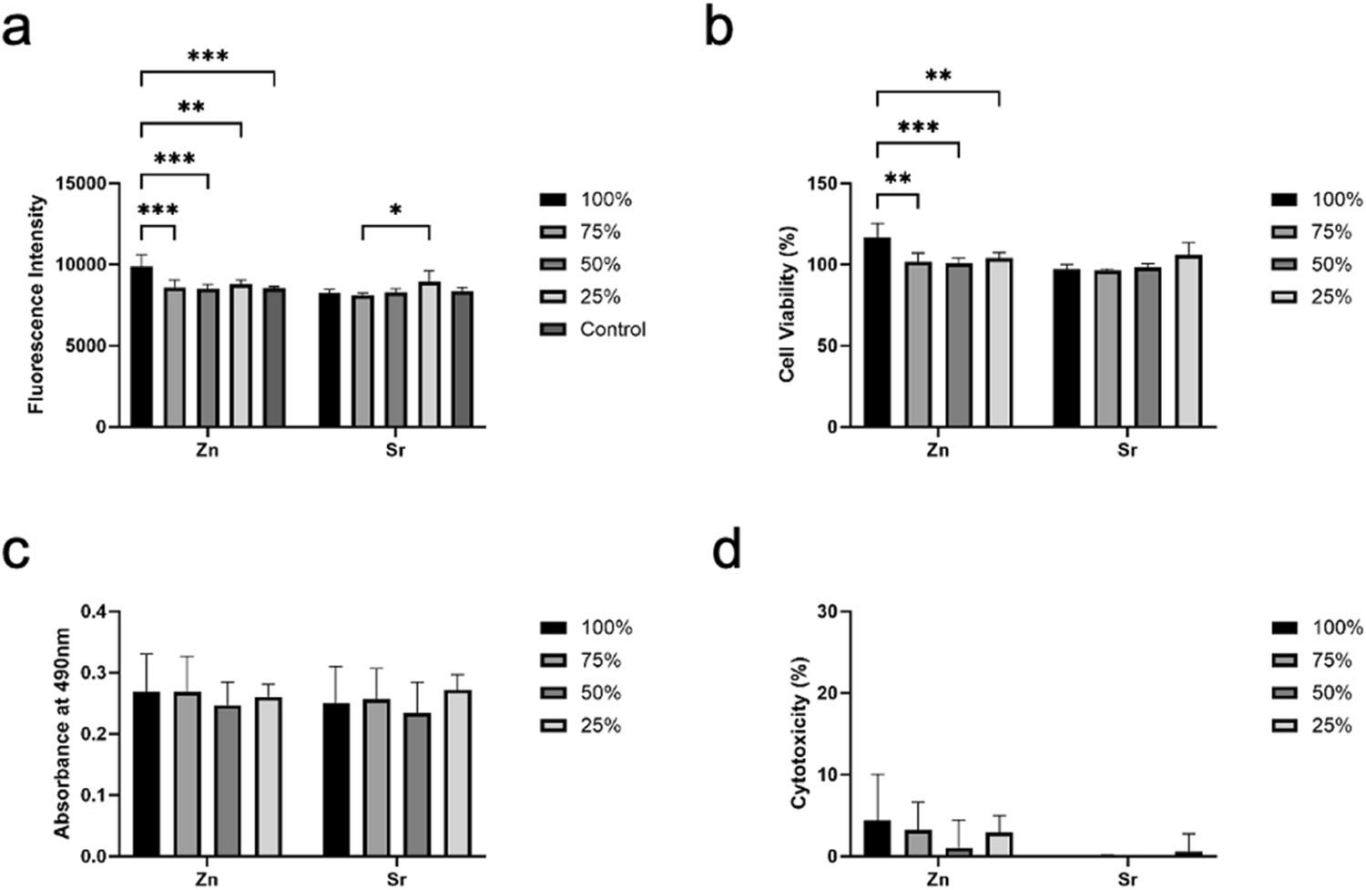
MC3T3-E1 cells were cultured with zinc or strontium phosphate glass extractions for 24 h. **a** The Alamar Blue assay was performed after 24 h, and fluorescence intensity was detected at 560/590 nm. The control groups were the cells cultured with complete growth medium. **b** The Alamar Blue cell viability was calculated by comparing the fluorescence intensity of extraction groups to the control groups. **c** The supernatant from each group was collected after 24 h and incubated with the LDH assay at room temperature for 30 min. The absorbance of LDH assay was measured at 490 nm. **d** The maximum LDH release was detected from each group by treating the samples with lysis buffer for 45 min. The percentages of cytotoxicity in each group were calculated by dividing the absorbance of LDH release by the absorbance of maximum LDH release

To study the cytotoxicity of active ion release, indirect contact assays were performed with extractions for MC3T3-E1 cells at first. Compared with the control groups, extractions from both zinc and strontium phosphate glass disks did not decrease the fluorescence intensity of the Alamar Blue assay (Fig. 2a). In addition, non-diluted Zn-Ti-Ca-Na-phosphate glass extractions significantly increased cell viability compared to diluted extractions (*P* < 0.01, Fig. 2b). This suggests that the ion release of zinc might be beneficial for cell growth. The LDH release was detected for extractions as well. There was no significant difference in LDH release between the extractions and control group (Fig. 2c). The percentage of cytotoxicity was calculated based on LDH release and maximum LDH release. The zinc or strontium phosphate glass extractions did not increase the percentage of cytotoxicity in this study (Fig. 2d).

**Fig. 3:**
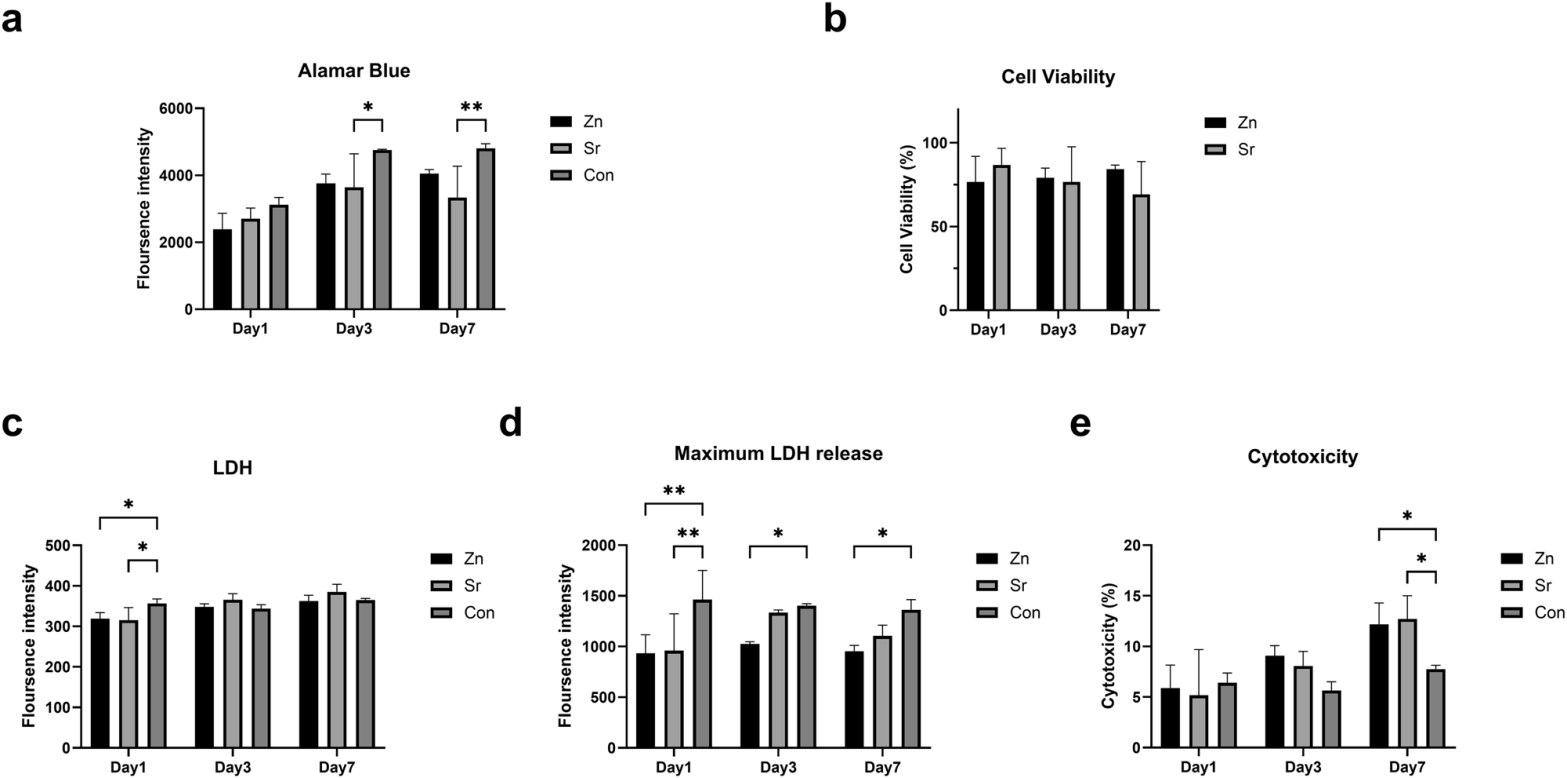
MC3T3-E1 cells were seeded on the top of zinc or strontium phosphate glass disks in 24 well plates for 1, 3, and 7 days. The control group was the cells cultured in the same 24 well plates with complete growth medium. **a** The Alamar Blue assay was performed after 1,3, and 7 days, and fluorescence intensity was detected at 560/590 nm. **b** The Alamar Blue cell viability was calculated by comparing the fluorescence intensity from zinc or strontium phosphate glass disc groups to the control groups. **c** The supernatant from each group was collected for each time point and then incubated with the LDH assay at room temperature for 10 min. The fluorescence intensity of LDH assay was measured at 560/590 nm. **d** The maximum LDH release at each time point was detected by treating samples with lysis buffer for 45 min. **e** The percentages of cytotoxicity in each group were calculated by dividing the fluorescence intensity of LDH release by the fluorescence intensity of maximum LDH release

**Fig. 4:**
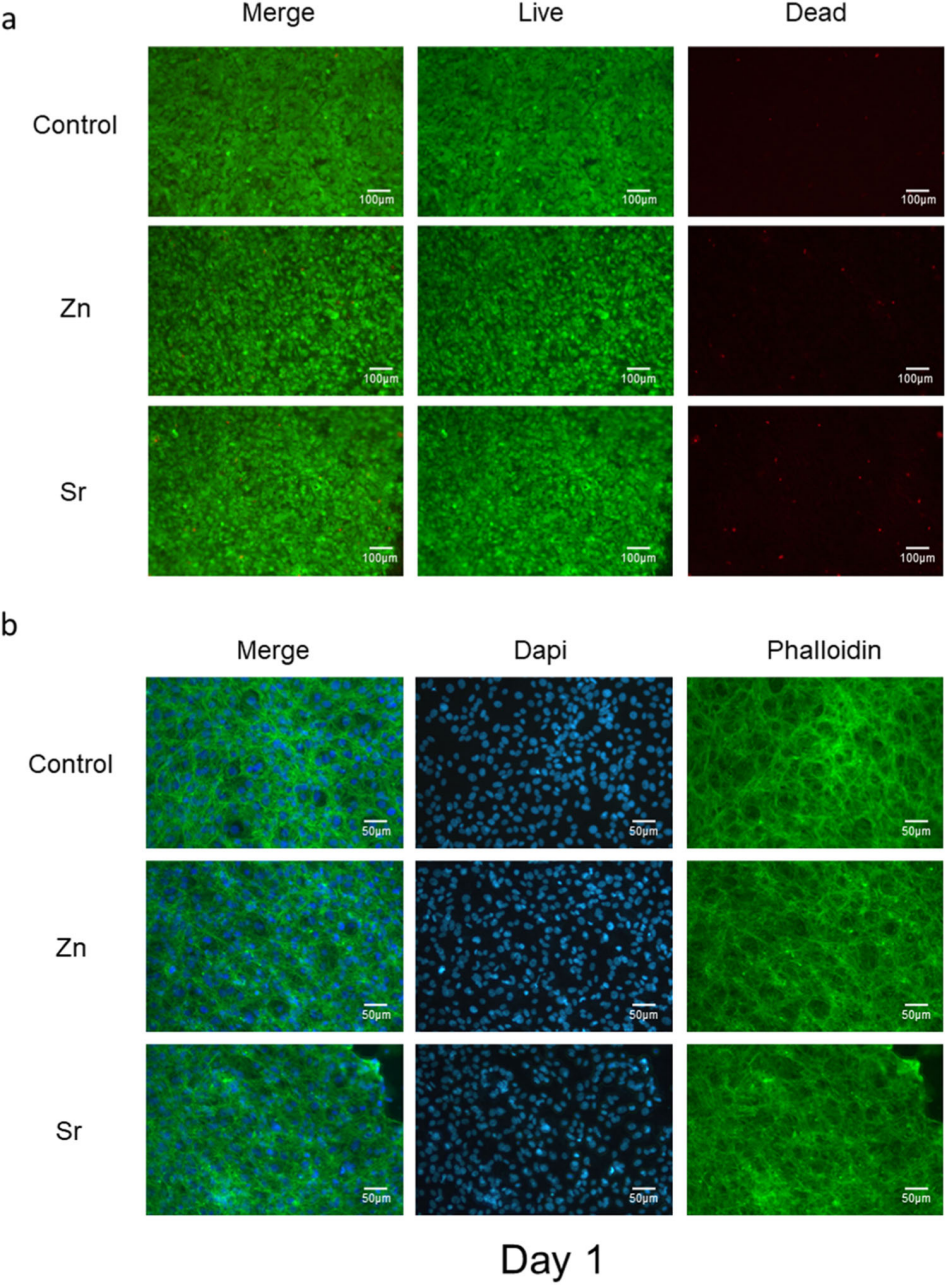
**a** The Live and Dead assay was performed with MC3T3-E1 cells that cultured on the top of glass disks after 1 day. The live cells were stained in green, and the dead cells were stained in red. **b** Samples in each group were fixed and permeabilized after 1 day. The nuclei were stained with Dapi in cyan and the cytoskeletons were labeled with phalloidin in green

Then, the MC3T3-E1 cells were seeded on the top of glass disks. The glass disks fit the bottom of 24 well plates, and we did not find any cells adherent to the bottom of the glass disks (Supplementary Fig. 1). No significant difference in the fluorescence intensity of Alamar Blue was found on the first day between all groups (Fig. 3a). The glass disks did not show a negative effect on the adherence of MC3T3-E1 cells either (Fig. 4a). Compared with the control group, the fluorescence intensity of the Alamar Blue assay decreased in Sr-Ti-Ca-Na-phosphate glass disks on Day 3 and Day 7. Meanwhile, there are no significant changes in cell viability between zinc and strontium phosphate glasses (Fig. 3b).

MC3T3-E1 cells cultured with phosphate glass disks did not increase the LDH release compared with the control group (Fig. 3c). However, the differences in total cell numbers were detected according to the maximum LDH release data (Fig. 3d). The maximum LDH release of zinc phosphate glasses is significantly lower than the control group, but the strontium phosphate glasses are not. Both zinc and strontium phosphate glass disks did not show effects on cytotoxicity until Day 7 (Fig. 3e).

The Live/Dead assays showed similar findings (Fig. 4a). Most cells were labeled with green fluorescence while few of them were in red. Besides, the morphology of MC3T3-E1 cells seeded on the top of glass disks after 1 day is shown in Fig. 4b. The cytoskeletons are in green, and nuclear are in cyan. The MC3T3-E1 cells can grow adherently on the surface of the glass disks until confluent (Supplementary Fig. 2 and Supplementary Fig. 3).

**Fig. 5:**
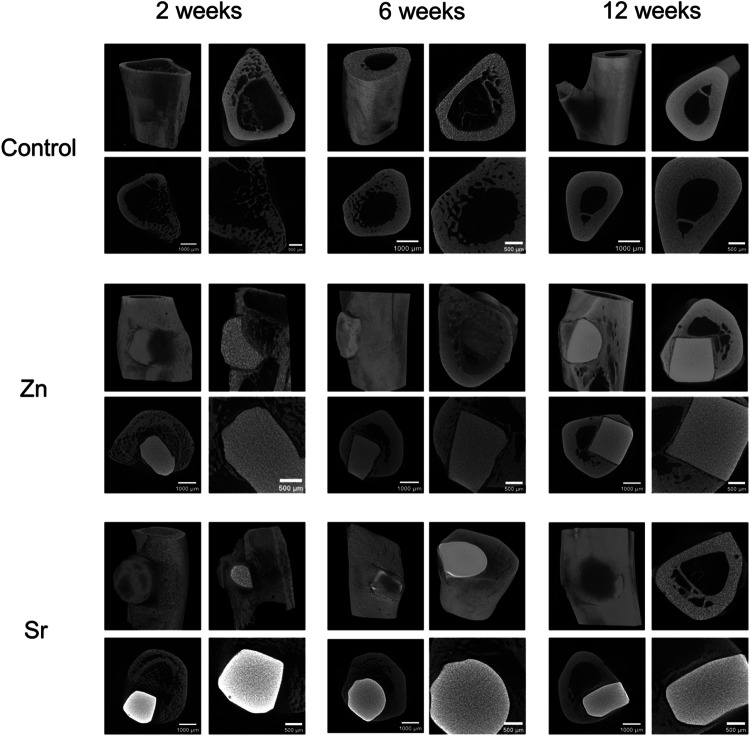
The Micro-CT analysis was performed at 2, 6, and 12 weeks after surgery. The images show the complete incorporation of zinc (Zn) or strontium (Sr) titanium-doped phosphate glasses into the newly formed bone and total bone healing in the control group

To further investigate the biocompatibility and biological effect of zinc and strontium phosphate glasses, the micro-CT analysis of rat tibiae with glass implants was conducted. The CT images have been reconstructed, based on a multi-dimensional projection into a grayscale image sequence (Fig. 5 and Supplementary Fig. 4). CT images were captured and analysed to detect neo-bone formation, the results have shown that compared to no-implant control, over the 12 weeks both strontium and zinc-based glass implants demonstrated favorable bone incorporation and longevity.

## Discussion

Two different types of glass disks were successfully made in this study. The glass disks were designed to cover the bottom of 24 well plates and subsequent in vitro experiments were conducted. In comparison with glass microspheres or fibers from our previous study, using glass disks made it easier to standardize the surface area for cell culture [15–17]. Additionally, there was cell adhesion on the top of glass disks without growth on the bottom (Supplementary Fig. 1). Therefore, the glass disks can be used as a model for the early study of cytotoxicity to analyse the effect of ion release and the surface morphology independently. However, it is worth noting that glass disks might have limited applications compared to glass microspheres or fibers for in vivo study. They are difficult to apply in complex and irregular bone defects and have a smaller surface area. Thus, glass particles were used for in vivo study.

The addition of Zn and Sr to phosphate glass had different effects on cell viability and cytotoxicity in vitro. Zinc oxide was shown to have a dose-dependent effect as undiluted Zn-conditioned media resulted in higher cell proliferation and lower cytotoxicity. Previous research suggests that the dose-dependent effect of zinc is due to the reduction in pH correlating with increased Zn release [15]. This study showed that lower LDH maximum release was recorded for 5 mol% of Zn phosphate glass which suggests that this concentration does not increase cytotoxicity and that it is suitable for culture in vitro. Higher concentrations of Zn have been shown to be cytotoxic, possibly due to further reduction in pH but also due to other factors which may need to be investigated [15]. The percentages of cell viability in all groups are ranging from 70% to 90%. This data is consistent with the live/dead assay, as few dead cells were observed on Day 1 (Fig. 4). Besides, as the cells confluent on Day 3 and Day 7, the number of live and dead cells both increased (Supplementary Fig. 2 and Supplementary Fig. 3).

Previous research demonstrated that phosphate glasses are promising for cell adherence and bone bonding [10, 18, 19]. We found that the highest concentration of zinc oxide significantly promotes cell viability, but Sr ions did not show a dose-dependent effect. This may be due to Strontium’s larger molecular size compared to calcium, so the addition of strontium to phosphate glass alters the composition and structure of the glass, which has been shown to reduce ion release of calcium ions [20]. Previous research suggests that strontium contributes more to osteoblast differentiation rather than proliferation which is beneficial in osseointegration [21].

Differences in maximum LDH release were found between the glass disks and the control group for the direct contact. As the MC3T3-E1 cells were seeded on the top of glass disks, the surface pattern might be one factor that led to lower maximum LDH release and cell numbers. The roughness of zinc phosphate glass disks and strontium phosphate glass disks are different according to the SEM images (Fig. 1). Research has shown that osteoblasts shown on rougher surfaces are less metabolically active than those grown on a flatter surface, which could explain the difference in cells grown on phosphate glass disks compared to the control [22]. Additionally, the difference in surface area might be another factor, as the zinc and strontium phosphate glass disks covered ~93% of the bottom in each well, which is smaller than the growth area of the control groups.

The in vitro findings of low cytotoxicity were supported by our in vivo study. The rat tibia defect animal model was established successfully (Fig. 5), and we observed bone regeneration over 12 weeks through micro-CT scanning. In the short term after surgery, we found that both zinc and strontium phosphate glass implants had good osseointegration. This finding shows that dense phosphate glass implants can provide good support and quickly repair bone structure, especially in the short term for replacing cortical bone. More importantly, we observed new cancellous bone formation surrounding the implants during this period, which might be beneficial for bone regeneration in longer term. However, it is important to acknowledge that due to the constrained sample size in this animal experiment, quantitative data suitable for statistical analysis were not attainable. Further experiments are required to explore further into this hypothesis.

## Conclusion

In conclusion, the investigation into titanium-doped phosphate glasses incorporating either 5 mol% zinc oxide or 17.5 mol% strontium oxide has yielded promising results for their application in bone tissue engineering. The bioactivity that these glasses possess, as proven by their superior biodegradability and active ion release capability and their biocompatibility quantified with the pre-osteoblast cell line MC3T3-E1 in both indirect and direct contact tests. The in vitro studies overall showed enhanced cell viability and negligible cytotoxicity, with zinc-doped phosphate glass extracts notably exhibiting a significant increase in cell viability. In addition, the in vivo studies utilizing a tibial defect model showed excellent bone incorporation and longevity over a 12-week period, highlighting the potential of these glasses for use in bone tissue engineering. Overall, the findings could suggest that titanium-doped phosphate glasses containing zinc oxide or strontium oxide hold great promise as biomaterials for advancing bone tissue engineering applications. Further research must be warranted to fully explore their potential in contributing to the development of innovative and effective strategies for bone regeneration.

## Supplementary Information

Supplementary Figures

## References

[1] Rahaman MN, Day DE, Bal BS, Fu Q, Jung SB, Bonewald LF, et al. Bioactive glass in tissue engineering. Acta Biomater 2011;7:2355–73. 10.1016/j.actbio.2011.03.016 21421084 10.1016/j.actbio.2011.03.016 PMC3085647

[2] Cannio M, Bellucci D, Roether JA, Boccaccini DN, Cannillo V. Bioactive glass applications: a literature review of human clinical trials. Mater (Basel) 2021;14:5440 10.3390/ma14185440 10.3390/ma14185440 PMC8470635 34576662

[3] Zamet JS, Darbar UR, Griffiths GS, Bulman JS, Brägger U, Bürgin W, et al. Particulate bioglass as a grafting material in the treatment of periodontal intrabony defects. J Clin Periodontol 1997;24:410–8. 10.1111/j.1600-051x.1997.tb00205.x 9205920 10.1111/j.1600-051x.1997.tb00205.x

[4] Pereira RDS, Menezes JD, Bonardi JP, Griza GL, Okamoto R, Hochuli-Vieira E. Histomorphometric and immunohistochemical assessment of RUNX2 and VEGF of Biogran™ and autogenous bone graft in human maxillary sinus bone augmentation: a prospective and randomized study. Clin Implant Dent Relat Res 2017;19:867–75. 10.1111/cid.12507 28608398 10.1111/cid.12507

[5] El-Ghannam A, Amin H, Nasr T, Shama A. Enhancement of bone regeneration and graft material resorption using surface-modified bioactive glass in cortical and human maxillary cystic bone defects. Int J Oral Maxillofac Implants 2004;19:184–91. 15101588

[6] Knowles JC. Phosphate based glasses for biomedical applications. J Mater Chem 2003;13:2395–401. 10.1039/b307119g

[7] Lakhkar NJ, Park JH, Mordan NJ, Salih V, Wall IB, Kim HW, et al. Titanium phosphate glass microspheres for bone tissue engineering. Acta Biomaterialia 2012;8:4181–90. 10.1016/j.actbio.2012.07.023 22835676 10.1016/j.actbio.2012.07.023

[8] Lee MJ, Kim MJ, Mangal U, Seo JY, Kwon JS, Choi SH. Zinc-modified phosphate-based glass micro-filler improves Candida albicans resistance of auto-polymerized acrylic resin without altering mechanical performance. Sci Rep. 2022;12:19456 10.1038/s41598-022-24172-y 36376540 10.1038/s41598-022-24172-y PMC9663707

[9] Khader A, Arinzeh TL. Biodegradable zinc oxide composite scaffolds promote osteochondral differentiation of mesenchymal stem cells. Biotechnol Bioeng 2020;117:194–209. 10.1002/bit.27173 31544962 10.1002/bit.27173

[10] Qaysi MA, Petrie A, Shah R, Knowles JC. Degradation of zinc containing phosphate-based glass as a material for orthopedic tissue engineering. J Mater Sci Mater Med 2016;27:157 10.1007/s10856-016-5770-x 27620740 10.1007/s10856-016-5770-x PMC5020113

[11] Ryu JH, Mangal U, Lee MJ, Seo JY, Jeong IJ, Park JY, et al. Effect of strontium substitution on functional activity of phosphate-based glass. Biomater Sci 2023;11:6299–310. 10.1039/d3bm00610g 37551440 10.1039/d3bm00610g

[12] Foroutan F, Kyffin BA, Abrahams I, Knowles JC, Sogne E, Falqui A, et al. Mesoporous strontium-doped phosphate-based sol-gel glasses for biomedical applications. Front Chem 2020;8:249 10.3389/fchem.2020.00249 32391313 10.3389/fchem.2020.00249 PMC7191082

[13] Lakhkar N, Abou Neel EA, Salih V, Knowles JC. Titanium and strontium-doped phosphate glasses as vehicles for strontium ion delivery to cells. J Biomater Appl 2011;25:877–93. 10.1177/0885328210362125 20219848 10.1177/0885328210362125

[14] AlQaysi M, Aldaadaa A, Mordan N, Shah R, Knowles JC. Zinc and strontium based phosphate glass beads: a novel material for bone tissue engineering. Biomed Mater 2017;12:12 10.1088/1748-605X/aa8346 10.1088/1748-605X/aa8346 28762960

[15] Salih V, Patel A, Knowles JC. Zinc-containing phosphate-based glasses for tissue engineering. Biomed Mater 2007;2:11–20. 10.1088/1748-6041/2/1/003 18458428 10.1088/1748-6041/2/1/003

[16] Vitale-Brovarone C, Novajra G, Lousteau J, Milanese D, Raimondo S, Fornaro M. Phosphate glass fibres and their role in neuronal polarization and axonal growth direction. Acta Biomater 2012;8:1125–36. 10.1016/j.actbio.2011.11.018 22134161 10.1016/j.actbio.2011.11.018

[17] Gupta D, Hossain KMZ, Ahmed I, Sottile V, Grant DM. Flame-spheroidized phosphate-based glass particles with improved characteristics for applications in mesenchymal stem cell culture therapy and tissue engineering. ACS Appl Mater Interfaces 2018;10:25972–82. 10.1021/acsami.8b05267 30011175 10.1021/acsami.8b05267

[18] Lin X, Chen Q, Xiao Y, Gao Y, Ahmed I, Li M, et al. Phosphate glass fibers facilitate proliferation and osteogenesis through Runx2 transcription in murine osteoblastic cells. J Biomed Mater Res A 2020;108:316–26. 10.1002/jbm.a.36818 31628823 10.1002/jbm.a.36818

[19] Mahato A, De M, Bhattacharjee P, Kumar V, Mukherjee P, Singh G, et al. Role of calcium phosphate and bioactive glass coating on in vivo bone healing of new Mg-Zn-Ca implant. J Mater Sci Mater Med 2021;32:55 10.1007/s10856-021-06510-0 33961158 10.1007/s10856-021-06510-0 PMC8105226

[20] Nguyen T-DT, Jang Y-S, Lee M-H, Bae T-S. Effect of strontium doping on the biocompatibility of calcium phosphate-coated titanium substrates. J Appl Biomater Funct Mater. 2019;17:2280800019826517 10.1177/2280800019826517 30803306 10.1177/2280800019826517

[21] Zhang W, Shen Y, Pan H, Lin K, Liu X, Darvell BW, et al. Effects of strontium in modified biomaterials. Acta Biomater 2011;7:800–8. 10.1016/j.actbio.2010.08.031 20826233 10.1016/j.actbio.2010.08.031

[22] Ball M, Grant DM, Lo W-J, Scotchford CA. The effect of different surface morphology and roughness on osteoblast-like cells. J Biomed Mater Res A 2008;86A:637–47. 10.1002/jbm.a.31652 10.1002/jbm.a.31652 18022800

