# Supplementary material for: Titanium-doped phosphate glasses containing zinc and strontium applied in bone regeneration
Source: J Mater Sci Mater Med. 2024 Jun 20;35(1):33. doi: 10.1007/s10856-024-06804-z (PMC11189983; doi:10.1007/s10856-024-06804-z)
Supplement: Supplementary file 1 — Supplementary Figures [file 10856_2024_6804_MOESM1_ESM.docx]

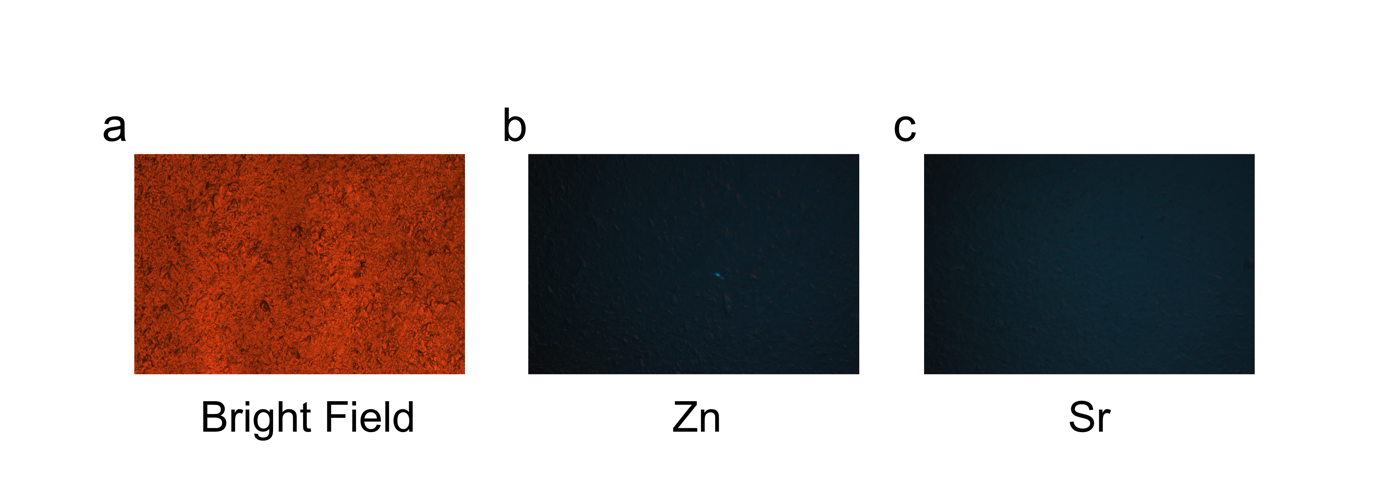


**Supplementary Fig.1** The bottom of glass discs was checked under fluorescence microscope. The MC3T3-E1 cells were seeded on the top of glass discs and stained with Dapi after 24 hours. (a) The typical bright field images of the bottom of zinc phosphate glass disc. (b) The typical fluorescence images of the bottom of zinc phosphate glass disc. (c) The typical fluorescence images of the bottom of strontium phosphate glass disc.


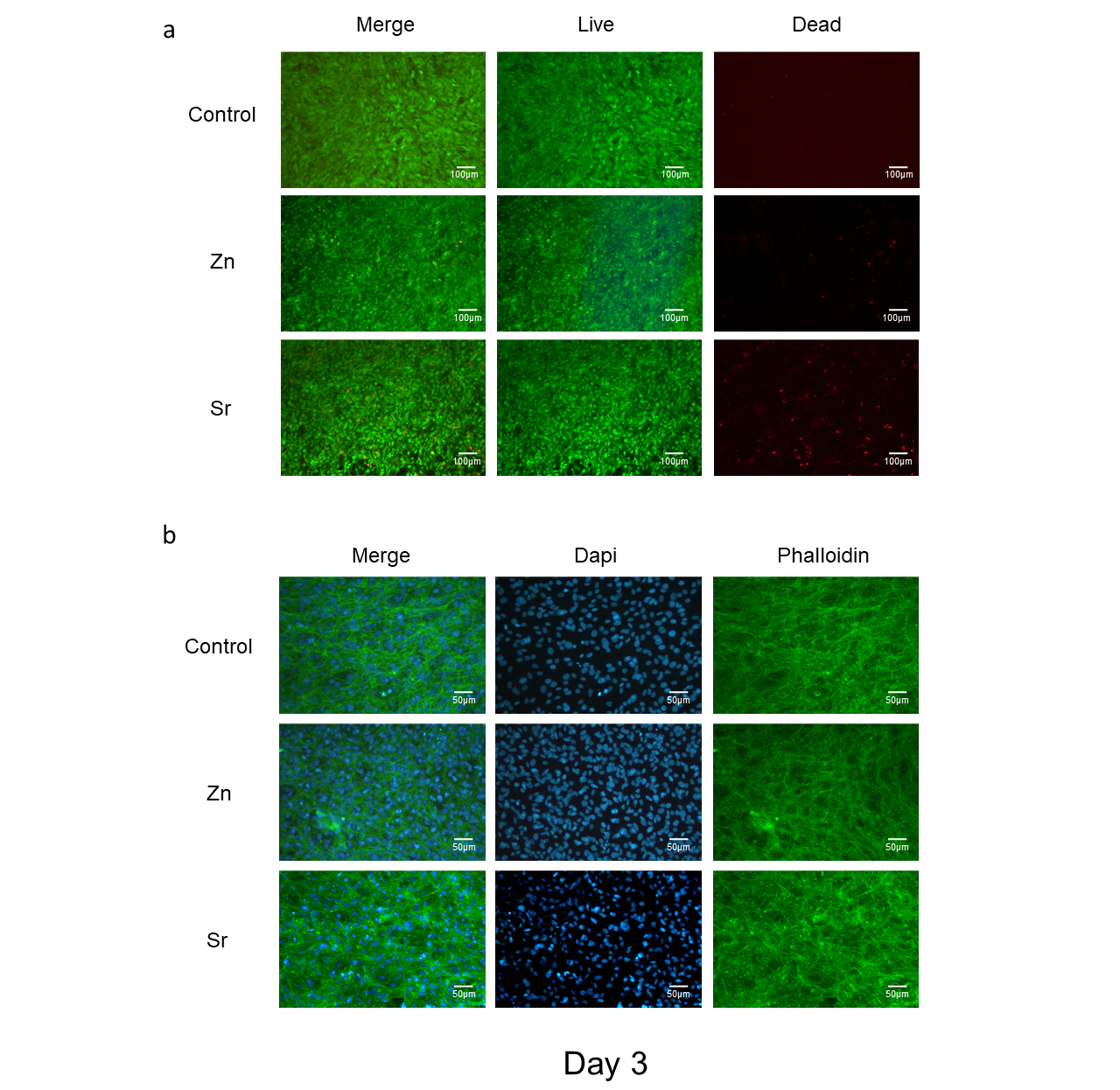


**Supplementary Fig.2** (a)The Live and Dead assay was performed with MC3T3-E1 cells that cultured on the top of glass discs after 3 days. The live cells were stained in green, and the dead cells were stained in red. (b) Samples in each group were fixed and permeabilized after 3 days. The nuclei were stained with Dapi in cyan and the cytoskeletons were labelled with phalloidin in green.


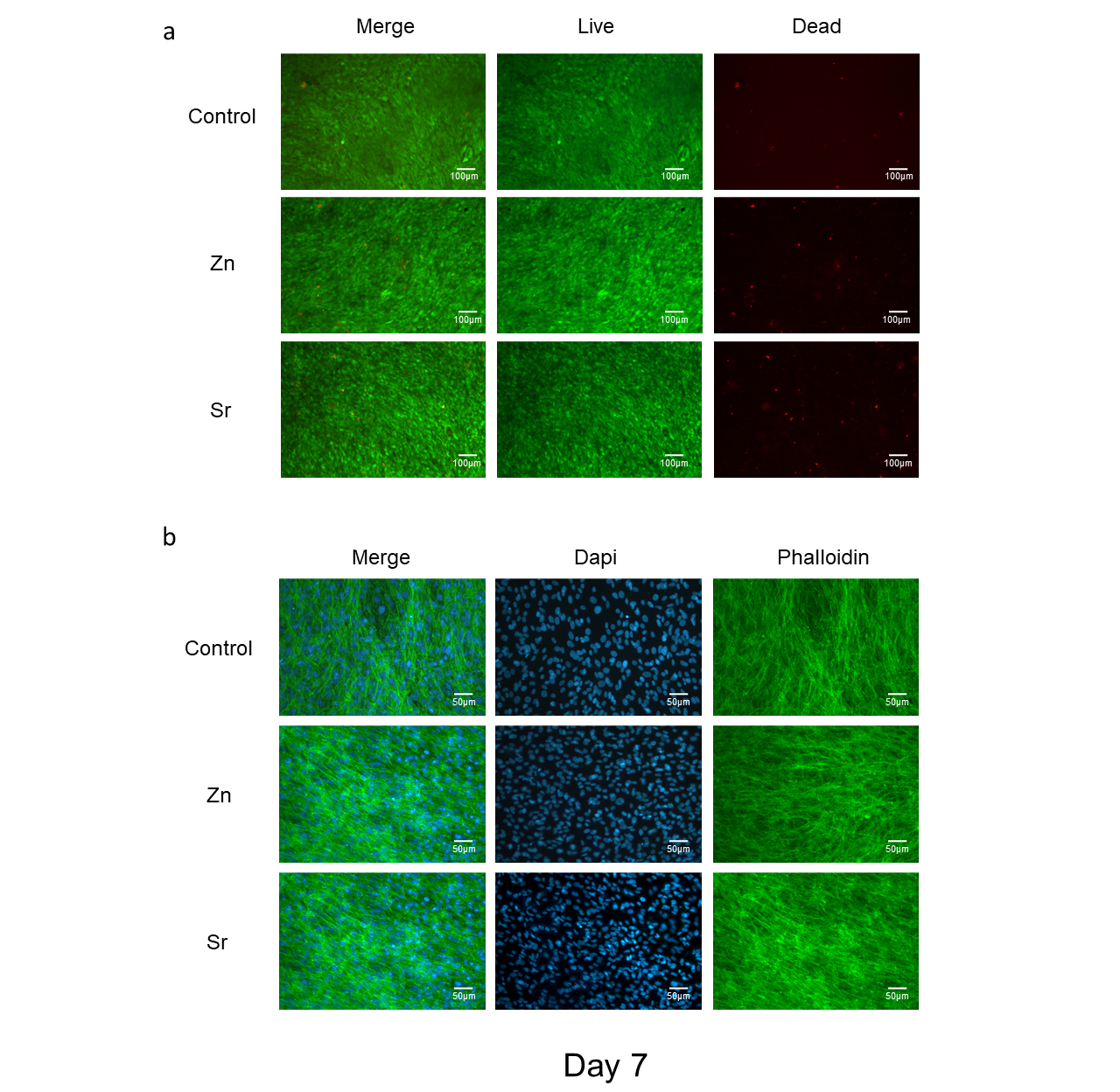
**Supplementary Fig.3** (a)The Live and Dead assay was performed with MC3T3-E1 cells that cultured on the top of glass discs after 7 days. The live cells were stained in green, and the dead cells were stained in red. (b) Samples in each group were fixed and permeabilized after 7 days. The nuclei were stained with Dapi in cyan and the cytoskeletons were labelled with phalloidin in green.


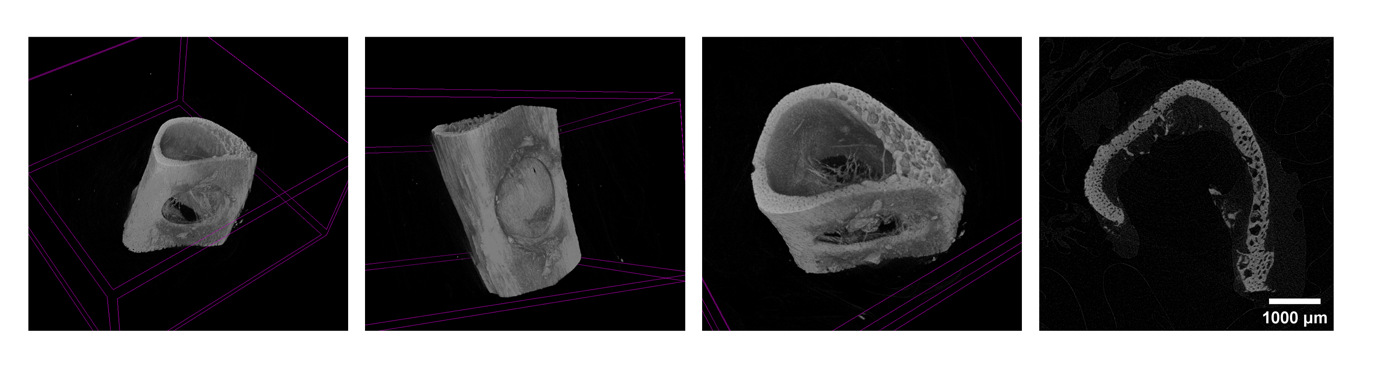


**Supplementary Fig.4** The Micro-CT analysis of the rat sacrificed immediately after the surgery.
